# 

**Published:** 1901-04-06

**Authors:** 


### The Hospital

A JOURNAL OF

THE MEDICAL SCIENCES AND HOSPITAL ADMINISTRATION,

INCLUDING

### A SPECIAL SECTION FOR NURSES.

IN TWO VOLUMES ANNUALLY.

EDITED BY

SIR HENRY BURDETT, K.C.B.,

AND

SOLOMON C. SMITH, M.D.

LIBRARY

SURGEON GENERAL'S OFFICE

FEB. 26 1902

177537.

VOLUME XXX.

April to September, 1901.

London:

PUBLISHED BY THE SCIENTIFIC PRESS (LIMITED), AT THE HOSPITAL BUILDING,
28 & 29 SOUTHAMPTON STREET, STRAND, W.C.

MDCCCCI.

W1
HO69X

### INDEX TO VOL. XXX., 1901.

*** Articles under the general heading *Hospital Clinics and Medical Progress* are distinguished by the initial (C.) for *Hospital Clinics*, and by (P.) for the rest of the series. (W.) distinguishes the *Institutional Workshop* articles, and (A.) the *Annotations.*

Abdomen, Gunshot Wounds of the (P.) 286
Abdominal Surgery, Progress in (P.), 286
Abdominal Viscera, Surface Anatomy of the (C.), 327, 343
Abscess of the Brain (C.), 146
Abscess of the Liver (P.), 348
Abscess, Otitic Extradural (P.), 117
Acne hypertrophica rosacea (P.), 134
Acromegaly, Visual Changes in (P.), 331
Adiposa dolorosa (P.), 432
Adiposity, Operation for (C.), 195
Advertising, the "Infamy" of, 174
Air Swallowing (P.), 182
Air Track through the Nose (C.), 261
Albuminuria, Cyclic, of Adolescence (P.) 83
Alcohol and the Hospitals (W.), 234, and *see p.* 272
Algiers, the Climate of (W.), 72
Alopecia (P.), 133
Amenorrhœa (P.), 163
Anæmia (C.), 63
Anæsthesia, Local (C.), 115
Anæsthetics, Progress in (P.), 230
Analgesia by Spinal Injection (P.), 84
Anastomotic Openings between Hollow Viscera (C.), 147
Aneurysm (P.), 433
Animal Parasites of the Skin (P.), 118
Animals on the Stage and Off, 69
Ante-mortem Invasion by Non-pathogenic Microbes (C.), 147
Antiseptics (P.), 65
Antityphoid Inoculation (C.), 428
Anuria, Post-operative (P.), 247
Apomorphine as a Hypnotic (P.), 217
Appendicitis (P.), 198, 333
Appendicitis, Fulminating (C.), 7
Architects, The, and the Medical Officers of Health, 324
Army Medical Corps, The, 57
Army Medical Service, The, 93
Army Medical Service, The Expert Committee on the (A.), 241
Arsenic, *see p.* 430
Arsenic and Alcohol, Sir T. Lauder Brunton on (C.) 98
Arsenic in Beer (C.), 79
Arsenic in Food, The Permissible Dose of (A.), 18
Arsenic in the Hair (C.), 23
Arsenical Poisoning, Chronic (C.), 247
Arterial Degeneration (C.), 213
Arterial Hypertonus and Arterio-Sclerosis (P.), 362
Arthritis, Pneumo-coccal (P.), 48
Ascites (P.), 193
Ascites, The Treatment of (C.), 193
Astigmatism, Corneal, The Operative Treatment of (C.), 162
Asylum Dysentry (C.), 45
Athletic Exercise, Severe, Effect of, on the Young (P.), 377
Atresia Auris Congenita (P.), 100
Atrophic Rhinitis and Ozæna (P.), 132
Atrophy, Acute Yellow (P.), 264
Aural Exostosis (P.), 116
Auricle, Left, Dilatation of the (C.), 161
Bactericidal Properties of Blood (P.), 215
Bacteriology, Progress in (P.), 132, 214, 330
Bacteriology of Skin Affections (P.), 118
Bacteriology and Water Examination (C.), 129
Balfour (Mr.) on Medical Science, 141
Bathing in Fevers (C.), 194
Bazin's Malady (P.), 149
Beer Bill, The, 1
Belfast Royal Victoria Hospital (W.), 201, and *see pp.* 221, 237
Blackwater Fever (P.), 167
Bladder, Extraperitoneal Rupture of the (P.), 378
Bladder and Ureters, Surgery of the (P.), 284, 314
Blastomycosis (P.), 431
Blood, Bacteriological Examination of the (P.), 330
Blood-Count, The (C.), 212
Bolingbroke Hospital, Wandsworth Common (W.), 185
Bond, The late Mr. Thomas (A.), 175
Book World (*Books, Magazines, and Pamphlets Reviewed*)
  Adams. *See Knight.*
  Bennett. The Present Position of the Treatment of Simple Fractures of the Limbs, 50
  Bennett. The Use of Massage in Fractures, 86
  Bernhard (trans. by Foster). First Aid to the Injured, 68
  Bland-Sutton. Tumous, Innocent and Malignant, 350
  Browden. *See Skrine*
  Burdett. Burdett's Hospitals and Charities, 1901, 200
  Cantlie. Plague, 400
  Carpenter. Syphilis of Children, 86
  Colbeck. Diseases of the Heart, 168
  Coolidge. Guide to Switzerland, 318
  Dewey. The No-Breakfast Plan and the Fasting Cure, 302
  Foster. *See Bernhard*
  Galt. Microscopy of the Starches, 200
  Greenwood. The Law relating to Poor Law Medical Service and Vaccination, 334
  Guide Books, Some Advertising, 318
  Hine. Practical Guide to the Public Health Acts, 302
  Keith. On Sanitary and Other Matters, 68
  Knight (edited by Adams). Self Educator in Chemistry, 350
  Local London, 334
  Macindoe. Sidmouth as a Health Resort, 318
  Medical Annual, 1901, 50
  Moore. Laboratory Directions for Beginners in Bacteriology, 68
  Parsons. Outlines for Dissectors, 168
  De Santi. Golden Rules of Aural and Nasal Practice, 350
  Skrine and Browden. The Tea We Drink, 86
  Surgical Regulations, Great Yarmouth Hospital, 50
  Vacher. Food Inspectors' Handbook, 350
  Wise. How to Avoid Tubercle, 86
  Woodhead. Report on Bacteriological Diagnosis, 400
Bottini Operation for Prostatic Obstruction, The (P.), 379
British Association, The, 389
British Medical Association, The (C.), 245-275, (C.), 295
Brominol (C.), 162
Brompton Hospital and Open-Air Treatment (A.), 257
Bubonic Plague at the Cape, 17
Cacodylic Acid (C.), 23
Calisthenics for the Early Riser (A.), 309
Cambridge Sanatorium (P.), 435
Cancer (P.), 197, 308; (P.), 431
Cancer, Progress in (P.), 134, 150, 346
Cancer of the Stomach (P.), 300
Carcinoma of the Duodenum (P.), 198
Carnegie (Mr.) and the Scottish Universities, 173
Cases from the Royal Hospital for Children and Women, London (C.), 411
Cell and the Molecule, The (A.), 409
Census, The (A.), 2, 94
Central Board of Health for London, A, 390
Cerebellar Hæmorrhage (P.), 233
Cheap Rides in Hot Weather (A.), 357
Chemistry of Nerve Degeneration, The (P.), 199
Chemists' Exhibition, 1901 (W.), 368
Chlorosis, 126
Cholesteatoma of the Temporal Bone (P.), 116
Christian Scientists and Life Assurance (A.), 209
Cirrhosis, Splenomegalic, of the Liver (P), 102
Civilian Surgeons in Time of War (C), 211
Civilisation and Disease (A.), 409
Cold Storage in Public Abattoirs (A.), 309
Colliery Explosion in South Wales, The (A.), 142
Collingridge, Dr., on Rats (A.), 34
Colonies, The, and the Child, 366
Colour Cure, The (A.), 325
Colour Problem, The (A.), 35
"Conjoint," Course of Study for the, 382
Conjunctival Sac, Bacteriology of the (P.), 332
Consulting Room Ethics (A.), 191
Convalescent Home on New Lines, A (W.), 337
Cornea and Sclera, Transplantations of (P.), 66
Coroner and his Jury, A, 76
Coroners and the General Public (A.), 3
Countess of Dufferin's Fund, *see p.* 321
County "Isolation" Hospitals (A.), 111
"Cramming" in Phthisis (C.), 193
Crofter, The Conservative (A.), 3
Croydon, The Health of, 94
"Cure" of Consumption, The (C.), 244
Cure of the "Incurable," The (C.), 113
Cyst, Echinococcus, of the Liver (P.), 347
Cyst, Ovarian, The Diagnosis of an (C.), 145
Cystitis (P.), 132
Cystoscopy in the Female (P.), 365
Cystostomy, Supra-Pubic, An Improved Method of Performing (P.), 364
Dangerous Trades, 51
Danysz's Microbe (C), 8
Deaf-Mute, Useful Hearing obtained in a (P), 25
Decadence of Races, The (A), 277
Delirium Tremens, Intra-Venous Saline Infusion in (C), 23
Dermatitis, Blastomycetic (P), 183
Dermatology, Progress in (P), 430
Diabetes, Progress in (P), 285, 298
Diagnosis by the Roentgen Rays (P), 433
Diaphragm, Rupture of the (P), 286
Diaphragmatic Abscess (P), 286
Diet, Some Current Absurdities in (C), 6
Dietetics (P), 265
Digestive Organs, Progress in Diseases of the (P), 181, 197, 249, 264
Diphtheria (P), 132, 330
Diphtheria, The Anti-Toxin Treatment of (C), 42
Diphtheria, A Contribution to the Ætiology of (C), 97
Diphtheria, Feeding in (C), 195
Dispensing (A), 309
Doyen of Hospital Secretaries, The, 255
Dropsy, Pathology of (P.), 362
Drug Eruptions—Arsenic (P.), 430
Drugs, The Strength of (A.), 292
Ductless Glands, Physiology of the (C.), 80
Ductus Arteriosus, Persistence of the (P.), 432
Duodenal Ulcer (P.), 363
Dying Depositions (C.), 147
Dysentery (P.), 249
Ear, Local Anæsthesia in the (P.), 25
Eclampsia, Puerperal (P.), 349
Eclipse Blindness (P.), 66
Ectopic Gestation (P.), 416
Eczema (P.), 118
Editor's Letter Box, 31, 55, 107, 139, 221, 237, 272, 321, 369, 405
Electricity in Treatment (A.), 341
Encephalopathy and Insanity from Recent Syphilis (P.), 196
Endocarditis, Gonorrhœal Ulcerative (P.), 433
Endocarditis, Malignant (P.), 378
Enterectomy and Intestinal Anastomosis (P.), 363
Enteric Fever in Campaigning, 276, and *see p.* 321
Epidermolysis Bullosa (P.), 134
Epilepsy, Treatment of (P.), 64
Epileptic Colony at Chalcote, The (W.), 287
Epiplyses, Separation of (C.), 394
Epistaxis, Profuse, Treatment of (P.), 433
Erythema Bullosum (P.), 133
Extra-Uterine Fœtation (C.), 311
Extra-Uterine Pregnancy, the Causes of (C.), 312
Facial Paralysis (P.), 217
Fallacy of Small Numbers, The (C.), 214
Fallopian Tube, Carcinoma of the (P.), 416
Fevers, The Open-air Treatment of (A.), 277
Fevers, Progress in (P.), 9, 27, 115, 148, 167, 315, 397, 417
Fibroids and Pregnancy (C.), 194
First Line of Defence, Our, 340
Fourth of July Tetanus (C.), 213
Fractures and Dislocations (P.), 180, 262
Free Baths (A.), 34
Fruit Wines, The Components of (A.), 77
Gall Stones (P.), 264
Gas; Ether; Chloroform (C.), 7
Gastric Ulcer (P.), 182, 197
Gastrotopsis (P.), 198
Gelatine Treatment of Aneurysm, The (C.), 359
General Surgery, Progress in (P.), 65, 180, 262
Genito-Urinary Surgery (P.), 364
Glances at the Hospitals (W.), 73, 90, 106, 337, 353, 404, 419
Gonorrhœal Iritis (C.), 45
Gonorrhœal Rheumatism (P.), 49
Gout (P.), 117
Gout and Plumbism (P.), 285
Gynæcology, Progress in (P.), 8, 163, 396, 414
Habitual Drunkards Bill, The, 125
Hæmophilia (P.), 117
Hæmorrhage from the Conjunctiva of an Infant (P.), 330
Hæmorrhage in Gastric Ulcer (C.), 45
Hæmorrhage, Post-mortem, Treatment of (C.), 62, and *see p.* 107
Hæmorrhagic Infection from a Species of Mucosus capsulatus (P.), 215

Hat-pin Swallowed by an Infant, A (C.), 131
Health in West Africa (C.), 395
Heart and Aorta, Surface Anatomy of the (C.), 427
Heart Failure from Over-strain (C.), 227
Heat Reflex (P.), 432
Hemiplegia and Sciatica, Electrical Treatment of (P.), 47
Hereditary Inebriety (A.), 111
Heredity Factor in Disease, The, 408
Hernia in Children (P.), 102
Hernia Strangulated in Early Infancy (C.), 162
Herpes Zoster (P.), 149
Herpes Zoster, Pathology of (P.), 47
Histogenesis of Ovarian Dermoids (P.), 397
Hospital Festivals, Meetings, etc., 12, 29, 55, 73, 89, 105, 122, 137, 153, 170, 186, 204, 220, 235, 250, 267, 305, 403
Hospital Sunday, 188
Hospital for the Unborn (A.), 18
Hospitals and Dispensaries in Burma (W.), 71
Hospitals, Enemies of the (W.), 201
Hospitals as Hot-beds of Infection (A.), 111
Hour Glass Stomach (P.), 301
Hydrocephalus, Internal, following Cerebro-Spinal Meningitis (P.), 47
Hygiene, A Great Experiment in (A.), 191
Hyperchloridria (P.), 182
Hypertrophy, Turbinal, Treatment of (C.), 213
Hysterectomy (P.), 9
Hysteria, Infantile (P.), 264
Ice Cream (A.), 127
Ichthyol in Eczema (C.), 99
Immunity in Fevers (P.), 315
Incipient Lunacy (A.), 241
Indian Asylums (A.), 19
Insane, General Paralysis of the (P.), 215
Insane, Story of the, from Year to Year (W.), 91, 106, 138, 155, 171, 271, 289, 435
Institutional Treatment of Disease, The (C.), 37
International Temperance Congress, The, 34
Intestinal Obstruction, Post-Operative (P.), 286
Intestinal Sand (C.), 63
Intestines, Progress in Surgery of the (P.), 363
Intussusception (P.), 364, (C.), 412
Intussusception, Treatment of (C.), 377
Iodine, Tincture of, in Infected Wounds (C.), 329
Iodine, Tincture of, in Dendritic Ulcer of Cornea (P.), 332
Jam Factory, A New (W.), 288
Joint Diseases, Classification of (P.), 49
Kernig's Sign (P.), 65
Kidney Disease, Physical Diagnosis in (P.), 246
Kidney Insufficiency and Operative Treatment (P.), 246
Kidney, Rupture of the, Treatment of (C.), 178
Kidney, Surgery of the (P.), 231
Kidneys, Treatment of Septic Inflammation of the (P.), 232
Koch, Professor, on Milk Tuberculosis, 291
Lameness, Intermittent, of Vascular Origin (P.), 377
Large Intestine, Cancer of the (P.), 363
Lead Poisoning (P.), 248
Lead Poisoning in Potteries, 51
League of Mercy, The (W.), 287
Leicester, New Isolation Hospital for (W.), 402
Leprosy (P.), 431
Leprosy, The Treatment of (C.), 99
Leucoma of the Vulva (C.), 261
Lewisham, Opening of St. John's Hospital (W.), 219
Lichen, Planus (P.), 119
Lithotomy, Lateral (P.), 315
Lithotomy, Suprapubic, My Experience of (C.), 61
Liver, Cirrhoses of the (P.), 249, 347
Liver, Contusions of the (P.), 348
Liver, Resection of the (P.), 347
Liver and Pancreas, Progress in Surgery of the (P.), 347
London and the Plague (A.), 95
London Hospital New Pathological Institute (W.), 266
London, The University of and Medical Education, 110
Lunacy and Crime (A.), 293
Lunacy Laws, The (C.), 395
Lunacy Work in London and New York (W.), 419
Lungs, Disease of the (P.), 10
Lungs, Vulnerability of the Apices of the, 190
Lupus (P.), 430
Lupus, Erythematosus (P.), 133, 430
Lupus, Vaccinal (P.), 183
Luxury in Surgery, 208
McKinley, The Attack on President (C.), 394
Madras Presidency, Civil Hospitals and Dispensaries in the (W.), 419
Malaria (P.), 148, 167, 417
Malaria, The Spread of (P.), 165
Malignant Growths with Cirrhosis (P.), 364
Marriage of the Unfit, The (A.), 34
Massage, The Use of, in the Treatment of Fractures (C.), 361
Mastoiditis (P.), 100
Match Maker, The Troubles of a, 87
Measles and Whooping Cough: Preventive Measures (P.), 165
Meckel's Diverticulum, *see p.* 363
Medical Acts, The Amendment of the (C.), 214
Medical Aid to the Women of India (W.), 321
Medical Council and Dr. Irvine, The, 158
Medical Education, 189, 381
Medical Guild and the Midwives, The (A.), 391
Medical Legislation, 372
Medical Man's Obligations, A (A.), 94
Medical Missionary, The (A.), 225, and *see p.* 273
Medical Missionary, The True (A.), 373
Medical Profession, The, 371
Medical Qualification, 382
Medical Schools, The, 383
Medical Statistics (C.), 428
Medical, Surgical, and Hygienic Exhibition, 169, 185, 220
Medicine and Midwifery (A.), 292
Medicine, Progress in (P.), 24, 362, 377
Melancholia, The Treatment of (C.), 376
Membrana-Tympani, Traumatic Rupture of the (P.), 26
Meningitis (P.), 232
Meningitis, Cerebro-spinal (P.), 330
Meningitis, Sporadic Cerebro-spinal (C.), 163
Mental Derangement, The Nature of (C.), 5
Mental Diseases, Training in (A.), 127
Mental Disorders following Influenza (P.), 84
Mental Disorders following Syphilis (P.), 101
Mental Disturbances after Operations on the Eye (P.), 67
Mercury, How to give (C.), 377
Methylene Blue as an Analgesic (P.), 217
Metropolitan Hospital Sunday Fund (W.), 303
Micro-organisms in Normal Tissues (P.), 133
Midwives, The Registration of (A.), 325
Migraine, Surgical Treatment of (P.), 199
Military Benevolent Fund, The (W.), 70
Milk—Is Raw Milk Injurious? (C.), 229
Milk, Pure (C.), 228
Mitral Stenosis (P.), 432
Modern Sociology, 51, 69, 87, 366, 380, 418, 434
Morphinomania, The Treatment of, (C.), 328
Moses and Tuberculous Meat (A.), 341
Motors (A.), 95
Movable Kidney (P.), 246
Muscle Albumin as Food, (C.), 359
Myasthenia Gravis (P.), 248
Mydriatics in Ophthalmology (P.), 67
Myosites Ossificans (P.), 117
Nævus (P.), 431
National Association for Supplying Medical Aid to the Women of India (W.), 321
National Education, 407
National Hospital, The, 207, 240 (W.), 250
Nerve Degeneration, The Diagnosis of (C.), 44, (P.), 199
Nervous System, Progress in Diseases of the (P.), 199, 217
Neurology, Progress in (P.), 47, 64, 232, 247, 263
Neuroses, Occupation (P.), 263
Neuroses of Childhood (P.), 83
New Appliances and Things Medical, 11, 103, 119, 135, 151, 199, 233, 265, 301, 333, 365, 379, 417
New Pathological Institute, Opening of a (W.), 254
Nordrach, The Treatment at (C.), 245
Nordrach-on-Dee Sanatorium, Banghory, near Aberdeen (W.), 152
Northampton General Infirmary (W.), 184, 335
Notes and News, 4, 20, 36, 60, 78, 96, 112, 128, 142, 160, 176, 192, 210, 226, 242, 258, 279, 294, 310, 326, 342, 358, 374, 392, 410, 426
Notification and Libel (A.), 3
Obstetrics, Progress in (P.), 348
Obstipation (C.), 312
Ocular Headaches (P), 47
Œsophagitis, Membranous (P.), 181
Œsophagus, Dilatation of the (P.), 182
Oh! those Newspapers (A.), 409
Open air Treatment in London (C.), 21
Open Shop, The (A.), 225
Operations by House Surgeons (A.), 391
Ophthalmology, Progress in (P.), 67, 331
Otitis Interna (P.), 26
Otitis Media, Acute (P.), 26
Otitis Media, Acute, Facial Paralysis in (P.), 27
Otology (P.), 25, 100, 116, 399, 414, 429
Our Lady Competitors, 355
Ovarian Cysts, Intra-peritoneal Rupture of the (P.), 164
Ovary as an Organ of Internal Secretion, The (P.), 396
Over-feeding of Infants, The (C.), 393
Pancreatitis (A.), 348
Pap-fed People, A (C.), 345
Paralysis Agitans (P.), 232
Paris and its Drains (C.), 161
Pathology, Animal and Vegetable (C.), 376
Pediatrics, Progress in (P.), 83, 102
Pericarditis, Suppurative (C.), 395
Peritonism (P.), 299
Peritonitis, The Pulse in (C.), 131
Peritonitis, Tubercular (P.), 286
Perspiration during Exertion (C.), 361
Persulphate of Soda, 67, and *see p.* 107
Pharmacomania (A.), 391
Pharyngeal Pouch, A (C.), 130
Placenta Prævia, The Treatment of (C.), 22
Plague (P.), 27; (C.), 229; (P.), 417
Plague, The Bacteriological Diagnosis of (C.), 163
Plague, Measures against (C.), 328
Plague, The Mysterious (A.), 127
Plantar Reflex (P.), 217
Polyneuritis, Syphilitic (P.), 217
Poor Lady, The, 75
Poplar Hospital, Opening of the New Wing (W.), 218
Population and Public Health, 109
Post-mortem Examinations, Unnecessary (A.), 209
Post-partem Hæmorrhage (P.), 348
Practical Departments (W.), 55, 123, 155, 272
Prefrontal Lobes, Disease of the (P.), 64
Pregnancy, Extra-uterine (P.), 164
Pregnancy, Primary Ovarian (P.), 349
Presystolic Murmurs, Apical (P.), 432
Prince of Wales's Hospital Fund for London (W.), 120
Prisons, Workhouses, Convents and Isolation Hospitals (A.), 357
Privy Council and Dr. Irvine, The, 142
Promotion by Merit (A.), 159
Prostate, Enlargement of the (P.), 379
Prostate, Enucleation of the (C.), 313
Prostate, Total Extirpation of the (C.), 413
"Providence" (A.), 159
Provident Dispensaries, 224
Pseudo-Appendicitis (P.), 333
Pseudo-Tuberculosis (C.), 129
Psoriasis (P.), 133
Psychiatry, Progress in (P.), 84, 101, 196, 215
Public-House Trusts (W.), 320
Purpura (P.), 117
Quackery, Legal Restrictions on (A.), 159
Railway Servants and Nervous Strain (A.), 373
Raynaud's Disease, The Treatment of (C.), 414
Raynaud's Phenomena (C.), 361
Recreative Reading, 58
Recruits, The Examination of (A.), 425
Rectal Feeding, The Nutritive Value of (C.), 22
Reflex Passage of Vesical Contents into the Ureters (P.), 284
Registrar-General's Return, The, 356
Renal Calculus (P.), 231
Renal Colic (P.), 284
Renal Pelvis and Ureters, Primary Tumours of the (P.), 314
Retention of Urine in the Child (C), 179
Rheumatism, Acute Infective (P.), 285
Rheumatism and Gout (P.), 48, 117
Rhinoscleroma (P.), 431
Richmond Union Infirmary (W.), 367
Rickets, The Pathogenesis and Treatment of (C.), 43 (P.), 84
Ringworm (P.), 118
Roentgen Ray Treatment of Skin Disease (P.), 183
Roof Conservatories on Town Houses (A.), 293
Royal College of Surgeons, The (A.), 191
"Rubbish" Tea (A.), 59
Ruchill Hospital, Glasgow (W.), 88
St. Andrew's Cottage Hospital (W.), 319
St. Catherine's Home for Cancer, Bradford (W.), 104
St. John and St. Elizabeth, The Hospital of (W.), 266
St. Thomas's Hospital (W.), 219
Saline Infusion in Puerperal Eclampsia (C.), 115
Salt Starvation in Epilepsy (C.), 314
Sanatoria for Consumption, 39, 323
Sanatoria: Who is to Pay? 423
Sanitary Certificates and Sea-Side Lodgings (A.) 257
Sanitary Mediævalism (A.), 118
Scarlet Fever (P.), 330
School Board Certificates (A.), 225
Sea Sickness (C), 245
Seeing, The Art of, 157
Self-Sacrifice in the Cause of Science (A), 425
Sensory Disturbances resulting from Cerebral Lesions (P), 199
Shop Assistant, The Case of the, 380, 401, 418, 434
Silk Stockings and Tin Poisoning (A), 175
Skin Diseases, Progress in (P), 118, 133, 149, 183
Sleeping on Duty (C), 179
Smoke Abatement (A), 3
Smoke Nuisances (A), 209
Sociology. *See Modern Sociology*
Spinal Cord, Degeneration of the (P), 248
Spinal Cord, Sub-acute Combined Degeneration of the (P), 64
Stammering (C), 329
State Medicine, Progress in (C), 82, 165
Stockport Infirmary Additions (W), 136
Stockport Isolation Hospital, The (A), 277
Stocks and Another *v.* Watson, 223
Stomach (Diseases of the Digestive Organs) (P.), 181
Stomach, Dilatation of the (P.), 182
Stomach, Progress in Surgery of the (P.), 300, 317
Stone in the Bladder (C.), 145
Stoppage of the Tear Passage (P.), 66
Street Cries (A.), 241
Street Pavements (A.), 357, and *see p.* 405
Street Sweeping by Skirts, 339
Student of Medicine, The, 16, 32, 55, 74, 124, 139, 156, 171, 206, 221, 238, 273, 289, 306, 322, 338, 353, 369, 388, 406, 421, 438
Subdural Hæmorrhage with Convulsions (C.), 161
"Substitution" in Dispensing (A.), 425

177157

Sugar-Free Milk (O.), 211, and *see p.* 230
Suicide, Attempted, by Drinking Jeyes' Fluid (O.), 229
Summer Heat, 256
Surgery, Abdominal (P.), 286
Surgery of the Bladder and Ureters (P.), 284, 314
Surgery, General, 65, 180, 262
Surgery, Genito-Urinary (P.), 364
Surgery of the Intestines (P.), 363
Surgery of the Kidney (P.), 231
Surgery of the Liver and Pancreas (P.), 347
Surgery, Progress of (P.), 25, 299, 314, 364, 379
Surgery of the Stomach (P.), 300, 317
Surgery of the Vermiform Appendix (P.), 333
Syphilis, Primary, State of the Inguinal Glands in (O.), 44
Syphilis, Spinal, Clinical forms of (P.), 65
Syringing the Ear (O.), 330
Teeth and Growth of Children, The (C.), 115
Tetanus (P.), 46
Therapeutic Notes, 67, 103
Thorax, Surface Anatomy of the (O.), 243, 259
Thrombosis, Otitic Lateral Sinus (P.), 116
Thyroid Treatment (P.), 432
Tobacco Heart, The (O.), 6
Tongue, Coated (P.), 182
Toothsome Sausage, The (A.), 325
Toxic Origin of Brain Disease (C.), 227
Tremor (P.), 64
Tub, The, in Obstetrics (O.), 195
Tubal Pregnancy, Pathology of (O.), 8
Tubercle Bacillus, Organisms resembling the (P.), 214
Tuberculides (P.), 149
Tuberculosis (P.), 82
Tuberculosis, British Congress on (A.), 19
Tuberculosis Congress, The, 239 (O.), 279
Tuberculosis, Intestinal (O.), 313
Tuberculosis of the Middle Ear (P.), 116
Tuberculosis, The Notification of, 307
Tuberculosis, Pulmonary, Early Diagnosis of (C.), 289
Tuberculosis, The Royal Commission on (A.), 373
Tuberculosis in the Zoological Gardens (O.), 413
Tumours complicating Pregnancy and Labour (P.), 349
Two Winter Pastimes (A.), 59
Typhoid (P.), 9
Typhoid Fever (P.), 24, (C.), 344, (P.), 397
Typhoid Water (C.), 344
Ulcer of the Stomach (P.), 317
University College Hospital (W.) 52, 122
University, The Functions of a (A.), 175
Ureter, Double (P.), 314
Ureteral Calculus simulating Vesical Calculus (P.), 284
Ureterectomy, Total (P.), 284
Uric Acid, Formation of (P.), 117
Urinary Stone Formation, Prevention of (P.), 314
Urotropine, The Ethics of Using (C.), 344
Urticaria (P.), 431
Urticaria Pigmentosa (P.), 134
Uterine Appendages, Palpation of the (O.), 21
Uterine Appendages, Inflammatory Affections of the (P.), 164
Uterine Displacements (P.), 8
Uterus, Cancer of the (P.), 316
Uterus, Fibromyomata of the (P.), 165
Uterus, Malignant Disease of the (P.), 165
Uterus, Multiple Fibroids of the (O.), 24
Uterus, Prolapse of the (P.), 397
Uterus, Rupture of the (O.), 261
Vaccinia (P.), 132
Vaccinia and Smallpox (O.), 114
Vaccinia, Variola, and Varicella (P.), 316
Vaccination, Dishonest (A.), 142
Varicella in Adults (C.), 131
Vermiform Appendix, Surgery of the (P.), 333
Vomiting, Recurrent, in Children (P.), 83
Whooping Cough, Measures against the Spread of (P.), 165
Whooping Cough, Results of a New Method of Treatment (O.), 375
Why do We Drink? (A.), 341
Why not a State Examination? (A) 142
Within the Hospitals (W.), 367
Woburn Cottage Hospital (W.), 351
Women, Medical Schools and Qualifications for,
Wooden Houses (A.), 19
Work for Lady Sanitary Inspectors (A.), 35
Workhouse Infirmary Management (W.), 352
"X"-rays, The (O). 177
Yellow Fever (P.), 45, 215

### THE SPECIAL SECTION FOR NURSES.

Across the Seas, 37, 55, 83
Action for Libel by a Hospital Nurse, 264
An Appeal for Trained Nurses in the Mission Field, 8
Appointments, 8, 21, 45, 60, 77, 84, 98, 109, 120, 139, 153, 167, 177, 191, 200, 214, 227, 242, 256, 268, 283, 295, 306, 322, 335, 343
Army Nurses and the South African Medal, 237
Asylum Workers; Annual Meeting of the Association, 122
Beyond the Seas, 236, 252, 288
Book and its Story, A, 44, 88, 126, 179, 280, 333
British Congress on Tuberculosis from a Nursing Point of View, The, 238
College Nursing, 59
Colonial Nursing Association, The, 107
Compulsory Registration of Midwives, The, 188
Co-operation Awakens, The, 72
County Council Lectures on Sick Nursing, 267
Crimean Nurses and the Lord Mayor, 211
Croydon Infirmary Crisis, 21, 335
Day in my District, A, 133
Day and Night in a Maternity Hospital, 291
Death in Our Ranks, 21, 34, 96, 108, 124, 177, 296
District Nursing in Spain, 45
East London Nurses at the Mansion House, 61
Echoes from the Outside World, 10, 24, 42, 62, 75, 85, 99, 113, 125, 140, 154, 165, 178, 190, 201, 212, 229, 240, 254, 269, 281, 294, 308, 319, 332, 345
Eight Months at the Imperial Yeomanry Hospital, Deelfontein, 33
"Elizabeth," A Sketch from Life, 293
English Nurses in a Train Attacked by Boers, 11
Entertainment to District Nurses, 305
Ethics of Nursing, 41
Everybody's Opinion, 11, 25, 47, 63, 76, 86, 100, 111, 124, 138, 152, 166, 176, 191, 201, 212, 227, 241, 255, 270, 282, 295, 309, 320, 334, 346
Examination Questions for Nurses, 9, 71, 124, 162, 189
Fashion Producing Disease, A, 303
Fishermen as Nurses, 211
Florence Nightingale, 86
Food Value of Milk, The, 266
Functions of the Week, 197
High Altitudes for Invalids, 174
Hints on the Practice of Massage, 38
Homes for Inebriates, 56
Home from the War: a Chat with an Army Reserve Sister, 137
How the Heat Cases were Treated in Roosevelt Hospital, New York, 248
How Smallpox was Nursed at the Belvedere Hospital, Glasgow, 121
Hospital Sunday Supplement, Special
Ilkley Hospital and Convalescent Home, The, 143
International Congress at Buffalo, New York, The, 23
Irish Nursing Question, 320
Lectures to Head Sisters, 6, 22, 36, 239, 253, 263 278
Lectures to Nurses on Medicine and Medical Training, 18, 54, 82, 106, 132, 160, 184, 208, 234, 314, 340
Life in a Navvy Hospital, 342
Local Government Board and Workhouse Infirmary Nursing Association, The, 276
Madonna of the Wards, 46
Management of Convalescent Homes, Hints on, 277
Maternal Charity and District Nurses' Home, 93
Medical Lectures to Nurses, 4, 30
Metropolitan Asylums Board and their Matrons, The, 84
Metropolitan Nursing Association, 73
New Issue at Newton Abbot, The, 220
Norwood Cottage Hospital, 77
Notes on News from the Nursing World, 1, 15, 27, 51, 65, 79, 91, 103, 115, 129, 143, 157, 169, 181, 193, 205, 217, 231, 245, 259, 273, 285, 299, 311, 325, 337
Notes and Queries, 13, 26, 49, 64, 78, 89, 102, 114, 127, 140, 155, 168, 180, 192, 203, 215, 230, 243, 257, 271, 284, 297, 310, 323, 336, 347
Novelties for Nurses, 43, 100
Nurse in the Mission Field, The, 316
Nurse Ward's Packing, 40
Nurse's Claim for Damages, A, 188
Nurses' Bookshelf, The, 12, 112, 212, 242, 266, 293, 330
Nurses' Co-operation, The, 86, 97, 164
Nurses of Derbyshire Royal Infirmary, The, 224
Nurses' Home at Richmond Infirmary, 136
Nurses of the Hospital for Consumption and Diseases of the Chest, 31
Nurses on Board the Hospital Ships of the Japan Red Cross Society, 95
Nurses at Marlborough House, 223
Nurses' Meals in Hospital, 151
Nurses of the Metropolitan Hospital, The, 250
Nurses of the North London Consumption Hospital, 315
Nursing at the Base Hospital in Natal, 39
Nursing in a Bavarian Hospital, 166
Nursing in the Coloured Home and Hospital, New York, 135
Nursing of Convalescents, On, 139
Nursing in Corea, 35
Nursing in a Cottage Hospital in North Germany, 249
Nursing at the Erie County Hospital, Buffalo, 58
Nursing at Friedrichshain Krankenhaus, Berlin, 5
Nursing at the Great Yarmouth General Hospital, 19
Nursing in Irish Workhouses, 306
Nursing "Jack Tar" at Greenwich, 173
Nursing of Lupus Patients at the London Hospital, 161
Nursing Malaria, Some Hints on, 175
Nursing in the Malay Settlements, 151
Nursing in Manila, 268
Nursing at Mooi River Hospital, 57
Nursing Plague and Enteric: Experiences of a Colonial Nurse, 150
Nursing Question in Irish Poor Law Infirmaries The, 110
Nursing in a Seminary in South Africa, 134
Nursing in Syracuse, New York, 199
Nursing of Tropical Dysentery, 331, 344
Nursing at Warmbad, Transvaal, 289
Nursing in Western Australia, 96
Ophthalmia Neonatorum, 302
Preparation of Patients for Anæsthetics and After Treatment, 328
Presentation and Garden Party at Llangarran, 279
Presentations, 8, 21, 34, 60, 77, 84, 120, 139, 167, 189, 202, 211, 228, 289, 344
Presentation of War Medals, The, 149
Queen Victoria's Jubilee Institute for Nurses, 200
Queen Victoria's Nurses, 69
Reading to the Sick, For, 13, 26, 48, 64, 78, 89, 102, 114, 127, 140, 155, 168, 180, 192, 203, 214, 230, 243, 257, 271, 284, 297, 310, 323, 336, 347
Reception by the Queen of the Nurses of the Royal National Pension Fund, 221
Reception of "Queen's" Nurses by Queen Alexandra at Marlborough House, 185
Relapsing Fever Wards, Arthur Road Hospital, Bombay, In the, 198, 210, 226
Resignation of Miss Griffiths, 268
Royal British Nurses' Association, 70, 149, 187
Sick Nurses for Shipboard, 290, 304, 317
Sick Quarters for Sick Nurses, 235
South African Medal Roll, The, 255
South London District Nursing Association, 109
Sterilization and Storage of Milk, The, 279
"Study what you most Affect," 292
Suggested Reforms in Military Nursing, 147, 163
Surgical Lectures to Nurses, Notes of, 68, 94, 118, 146, 172, 196, 220
To and From the Front, 74
Through an Epidemic, 20
Training at Queen Charlotte's Lying-in Hospital, The, 119
Travel Notes, 14, 50, 60, 90, 112, 128, 142, 156, 164, 177, 187, 204, 216, 244, 258, 272, 298, 324, 348
Typical Patients, 32
University College Hospital Ladies' Association, The, 108
View Day at "Barts," 100
Visit to an American Sanatorium, A, 262
Visit to a Cingalese Insane Asylum, 7
Visit to No. 5 General Hospital, Wynberg, 328
Visit to a Quaker Insane Asylum in Pennsylvania, 209
Wants and Workers, 23, 48, 98, 108, 124, 344
War Nurses at Southampton, 341
Ward Washing Day in a Bombay Hospital, 46
West Ham Typhoid Epidemic, 123

Printed by SPOTTISWOODE & CO. LTD., New Street Square, E.C.; and Published by the Proprietors, THE SCIENTIFIC PRESS, LIMITED, at 28 & 29 Southampton Street, Strand, London, W.C.

### NOTICE TO CORRESPONDENTS.

All MSS., Letters, Books for Review, and other matters intended for the Editor should be addressed The Editor, "The Hospital," The Hospital Building, 28 & 29 Southampton Street, Strand, W.C.

The Editor cannot undertake to return rejected MS., even when accompanied by stamped directed envelope.

**Notice to Advertisers and Subscribers.**

All Advertisements, Orders for Copies of The Hospital, and Business Communications should be addressed to **THE MANAGER (Not to the Editor),** The Hospital, 28 & 29 Southampton Street, Strand, London, W.C.

The Cost of Subscription, post free, is as follows:—Per week, 2½d.; per quarter, 2s. 8d.; per half-year, 5s. 3d.; per annum, 10s. 6d. Foreign:—Per annum, 15s. 2d., payable, in all cases, in advance.

**NOTICE.—Vol. XXIX. of "The Hospital" (October, 1900, to March, 1901), in handsome cloth binding will shortly be on sale at the Office of this Journal, price, 7s. 6d. Cases for binding the Numbers contained in this Volume 1s. 6d. Index to Vol. XXIX., 2½d., post free.**

**The Monthly Part for April is now ready. Price 6d., by post 8d.**

### Notes and News.

### The Student of Medicine.

### APPOINTMENTS.

**Miss Annie M. S. Anderson**, M.D.Lond., appointed Honorary Physician to the Manchester Clinical Hospital. **W. E. Audland**, L.R.C.P.Lond., M.R.C.S.Eng., appointed Certifying Factory Surgeon for the Wellingborough District of Northants. **George H. Ayres**, L.R.C.P., L.R.C.S.Irel., appointed Certifying Factory Surgeon for the Tarporley District of Cheshire. **N. T. Brewis**, M.B., L.R.C.S.Edin., appointed Assistant Gynæcologist to the Edinburgh Royal Infirmary. **H. H. Broome**, M.B., C.M., appointed Senior Demonstrator of Anatomy at Owens College, Manchester. **William Cotton**, M.A., M.D., D.P.H., appointed Medical Officer H.M. (local) Prison, Bristol. **Joshua J. Cox**, M.D. Edin., appointed Honorary Physician to the Manchester Clinical Hospital. **Wilfrid Glegg**, M.D., M.R.C.P.Edin., appointed Senior Resident Medical Officer to the Throat Hospital, Golden Square, London, W.

King's College Hospital.—The following have been appointed Resident Medical Officers for the present six months: Senior House-Physician, **F. B. Jefferies**, M.R.C.S., L.R.C.P. Junior House-Physician, **W. L. Stuart**, M.R.C.S., L.R.C.P. Assistant House-Physician, **C. E. Bulteel**, M.R.C.S., L.R.C.P. House-Surgeons, **R. S. Corke**, M.R.C.S., L.R.C.P.; **A. Edmunds**, M.R.C.S., L.R.C.P.; **F. C. Carle**, M.R.C.S., L.R.C.P. House-Accoucher, **E. L. Saunders**, M.R.C.S., L.R.C.P. Assistant House-Accoucher, **W. W. Maxwell**, M.R.C.S., L.R.C.P.

